# The rapidly emerging public health threat of rabies in Timor-Leste, 2024–2025

**DOI:** 10.5365/wpsar.2025.16.3.1332

**Published:** 2025-09-22

**Authors:** Filipe de Neri Machado, Joanita Bendita da Costa Jong, Florindo P Gonzaga, Felisiano da Conceição, Anthony DK Draper, Mateus Pinheiro, Frederico Bosco Alves dos Santos, Noel Gama Soares, Mariano da Silva Marques, Marcelo Amaral Mali, Aloto Ximenes Belo Amaral, Benigna Veneranda da Costa Amaral, Nazario Barreto dos Santos, Adriano Barbosa, Livia Natalia Babo, Joshua R Francis, Merita Antonia A Monteiro, Nevio Sarmento

**Affiliations:** a Instituto Nacional de Saúde Pública de Timor-Leste, Comoro, Timor-Leste.; b Ministry of Health; Ministry of Agriculture, Livestock, Fisheries and Forestry, Government of Timor-Leste, Comoro, Timor-Leste.; c Directorate for Disease Prevention and Control, Ministry of Health, Lahane, Timor-Leste.; d National Veterinary Diagnostic Laboratory, Dili, Timor-Leste.; e Menzies School of Health Research, Charles Darwin University, Bidau Lecidere, Timor-Leste.; f National Centre for Epidemiology and Population Health, Australian National University, Canberra, Australian Capital Territory, Australia.; g Centre for Disease Control, Northern Territory Government Department of Health, Darwin, Northern Territory, Australia.; h Hospital Nasional Guido Valadares, Bidau, Timor-Leste.

Rabies is a public health concern in over 150 countries and territories, mainly in Africa and Asia, (*1*) and causes over 59 000 deaths per year. (*2*) The rabies virus is usually transmitted to humans through the bite or scratch of an infected animal. In low- and middle-income countries, most human cases result from dog bites, but the virus may also be transmitted by other animals. (*3*) If symptoms of rabies develop, the disease is almost always fatal, usually within days or months. A One Health approach is essential for the prevention of rabies. (*1*) It can be prevented through mass dog vaccination and through the provision of post-exposure prophylaxis (PEP) with rabies vaccine and rabies immunoglobulin (RIG) as soon as possible after potential exposure to the virus. (*4*)

Timor-Leste is a resource-limited nation of 1.4 million people (*5*) that shares a land border with Indonesia. The country was considered rabies-free until the first human and animal cases were detected in March 2024 in the Special Administrative Region of Oecusse-Ambeno (Oecusse). (*6*) Since then, it has been detected in dogs and humans in a growing number of municipalities. Any person in Timor-Leste who is bitten or scratched by an animal that could potentially transmit the rabies virus should be assessed for rabies exposure to determine their need for PEP. Access to vaccines and RIG should be increased by strengthening vaccine supply and integrating PEP into routine immunization programmes as well as primary and secondary health care, while increasing public awareness of the risk of rabies following dog bites. This paper briefly describes the evolving rabies situation in Timor-Leste as of July 2025 and offers lessons for other nations in the Indo-Pacific region where rabies may become endemic.

## Human rabies in Timor-Leste

**Fig. 1:**
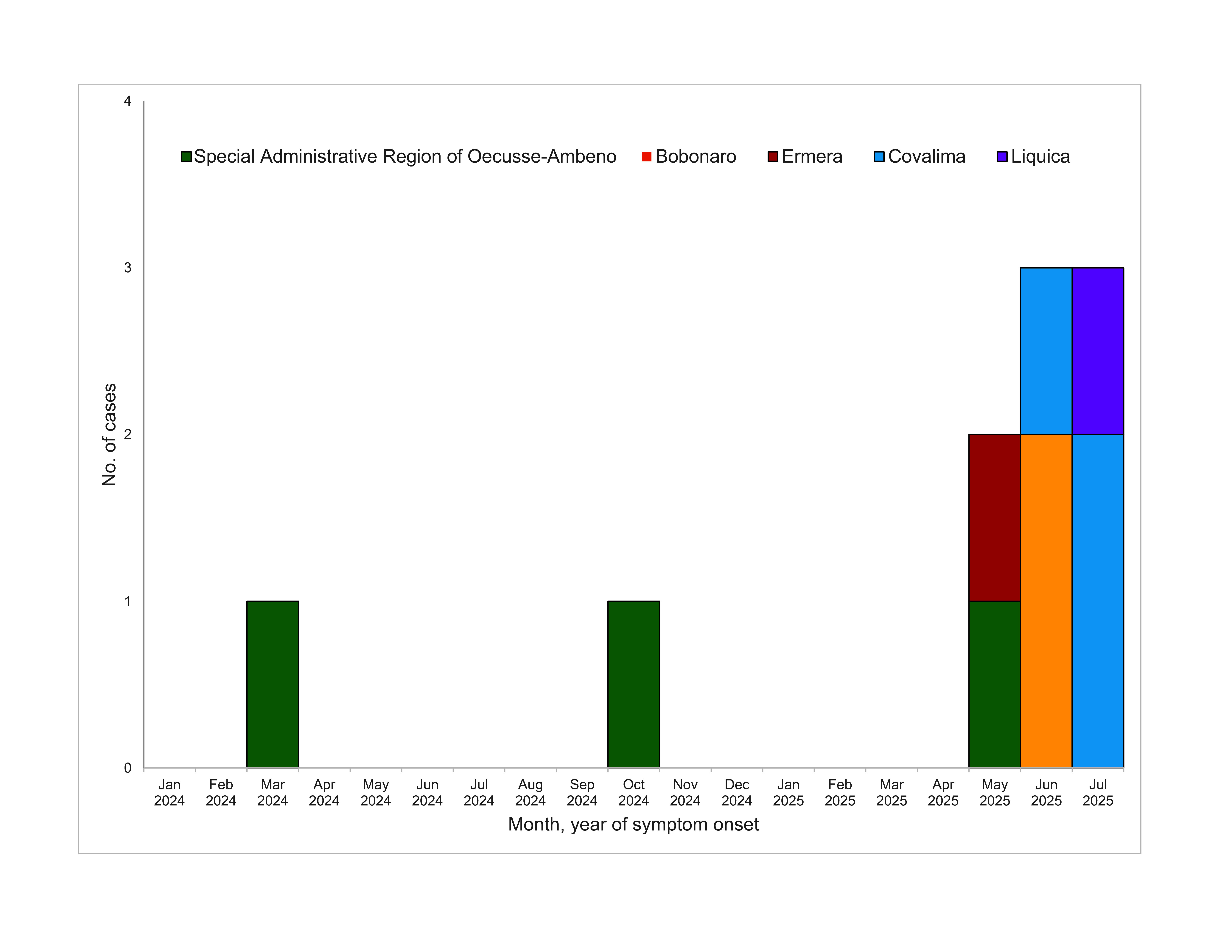
Epidemic curve of confirmed human rabies cases by municipality and month of symptom onset, Timor-Leste, 1 January 2024–31 July 2025

Between 1 January 2024 and 31 July 2025, 10 laboratory-confirmed cases of human rabies were reported in residents of five municipalities in Timor-Leste: Bobonaro, Covalima, Ermera, Liquica and Oecusse (**Fig. 1**). All cases were fatal. The median age was 21 years (range 2–54 years), and six (60%) were male. The median incubation period from rabies exposure to symptom onset was 96 days (range 85–251 days). On 16 June 2025, in response to the increasing number of cases, Timor-Leste declared rabies a public health emergency in the country. (*7*)

[**insert** Figure 1]

Between 1 April 2024 and 7 July 2025, 1987 cases of people being bitten by dogs were reported in Timor-Leste. Of these, 99 (5.0%) were bitten by dogs that tested positive for rabies. The dog bites were classified according to national guidelines for level of severity as follows: 72 (3.6%) were Category I (licks on intact skin); 835 (42.0%) were Category II (soft bite – skin bruised but not bleeding); and 965 (48.6%) were Category III (severe bites or any bat bites). (*8*) The exposure category was not recorded for 115 (5.8%) cases. The number of vaccine doses (RABIVAX-S, Serum Institute of India, Pune, India) administered is shown in **Table 1**.

**Table 1:** Number of rabies vaccine doses given to dog-bite victims, Timor-Leste, 1 April 2024 to 7 July 2025

Number of rabies vaccine doses	All dog-bite victims, *n*(%)	Bite victims of rabies-positive dogs, *n*(%)
**1**	**376 (18.9)**	**26 (26.3)**
**2**	**494 (24.9)**	**11 (11.1)**
**3**	**525 (26.4)**	**19 (19.2)**
**4**	**420 (21.1)**	**38 (38.4)**
**Unknown vaccination status**	**172 (8.7)**	**5 (5.1)**
**Total**	**1987 (100)**	**99 (100)**

Only 248 (12.5%) of all dog-bite victims and 52 (52.5%) of victims of rabies-positive dogs received RIG.

## Animal rabies in Timor-Leste

As cases of rabies increased in the neighbouring Indonesian province of Nusa Tenggara Timur in May 2023, (*9*) the Government of Timor-Leste launched a public awareness campaign and, in January 2024, began an intensive and ongoing mass vaccination programme for dogs, cats and monkeys. As of 7 July 2025, the rabies vaccine had been administered to 52 524 animals: 48 745 dogs, 3530 cats and 249 monkeys. (*10*)

A surveillance programme for animal rabies in Timor-Leste is ongoing. As of 22 July 2025, 140 animals had tested positive for rabies: 134 dogs, five goats and one pig. Animal detections were reported in five of the 13 municipalities, all in the western part of the country: Aileu, Bobonaro, Covalima, Ermera and Oecusse.

Veterinary surveillance officers have adopted a One Health approach to their response activities. Upon notification of dog-bite incidents or confirmation of rabies-positive animals, veterinary officers promptly inform the health-care services to ensure that potentially exposed individuals are assessed and, if appropriate, receive PEP.

## Discussion

Human and animal rabies cases have spread in Timor-Leste following the first human case in March 2024. Positive animal cases have been detected in five municipalities, and there will likely be further spread to other municipalities. Dog vaccination programmes and enhanced surveillance should continue in all municipalities in Timor-Leste to decrease the risk of transmission.

Any person in Timor-Leste who is bitten or scratched by an animal with the potential to transmit rabies (especially dogs, bats, monkeys and cats) should wash the wound immediately and present to a health post, health centre or hospital for rabies PEP assessment. When administered directly after potential exposure, rabies PEP is extremely effective in preventing infection and fatality.

It is important that human rabies vaccines and RIG are accessible in all areas of Timor-Leste, as animals with rabies are likely to continue moving eastward and into municipalities that have not yet been affected. Vaccines and RIG may be in limited supply in remote areas, and access should be ensured to reduce logistical barriers to immediate PEP administration following potential exposure. Mass dog vaccination should also continue.

We observed that only a small proportion of people bitten by dogs received the recommended full course of PEP doses. This could be due to difficulty in accessing health care or a lack of understanding of the risks from dog bites, given that Timor-Leste was until recently rabies-free. Increasing and maintaining high public health awareness through targeted, locally relevant health promotion is critical. Lessons from Timor-Leste may be applied to other nations in the Indo-Pacific region where similar challenges may exist.
